# Do funding applications where peer reviewers disagree have higher citations? A cross-sectional study.

**DOI:** 10.12688/f1000research.15479.2

**Published:** 2018-10-15

**Authors:** Adrian G. Barnett, Scott R. Glisson, Stephen Gallo

**Affiliations:** 1 Institute of Health and Biomedical Innovation & School of Public Health and Social Work, Queensland University of Technology, Brisbane, QLD, 4059, Australia; 2 American Institute of Biological Sciences, Reston, Virginia, VA 20191, USA

**Keywords:** meta-research, research funding, peer review, citations, research impact

## Abstract

**Background**: Decisions about which applications to fund are generally based on the mean scores of a panel of peer reviewers. As well as the mean, a large disagreement between peer reviewers may also be worth considering, as it may indicate a high-risk application with a high return.

**Methods**: We examined the peer reviewers' scores for 227 funded applications submitted to the American Institute of Biological Sciences between 1999 and 2006. We examined the mean score and two measures of reviewer disagreement: the standard deviation and range. The outcome variable was the relative citation ratio, which is the number of citations from all publications associated with the application, standardised by field and publication year.

**Results**: There was a clear increase in relative citations for applications with a better mean. There was no association between relative citations and either of the two measures of disagreement.

**Conclusions**: We found no evidence that reviewer disagreement was able to identify applications with a higher than average return. However, this is the first study to empirically examine this association, and it would be useful to examine whether reviewer disagreement is associated with research impact in other funding schemes and in larger sample sizes.

## Introduction

Winning funding is an important stage of the research process and researchers spend large amounts of their time preparing applications ^1^. Applications are typically assessed using relatively small panels of 3 to 12 peer reviewers, sometimes including external reviewers with additional expertise, which is similar to the journal process of editors and reviewers. Given the importance of funding processes to researchers’ careers and the progress of science, there is surprisingly little research on whether funding systems reliably identify the best research. A recent literature review found there are many unanswered questions in funding peer review, and concluded, “there is a need for open, transparent experimentation and evaluation of different ways to fund research” ^2^. An earlier systematic review similarly concluded that studies to examine the accuracy and soundness of funding peer review are “urgently needed” ^3^. Whilst a systematic review of innovations focused specifically on studies aiming to improve the effectiveness and efficiency in peer review funding found only eight studies and called for more studies of peer review ^4^.

The majority of funding systems rank applications using the mean score from the review panel, and award funding from the highest to the lowest ranked applications, stopping when the budget is exhausted (exceptions are sometimes made for applications below the funding line because of national research priorities). An interesting recent idea is that an application’s mean score may not be the only statistic worth considering, and that the standard deviation in peer reviewers’ scores may also be a useful statistic for ranking applications ^5^. A zero standard deviation means all panel members gave the same score. Larger standard deviations indicate more disagreement between panel members, and this disagreement may be useful for identifying high-risk research that may also have a higher return. A related alternative funding system is to fund all applications where reviewers agree on a high score, and then allocate the remaining budget at random where the reviewers disagreed but some reviewers gave the application a high score ^6^.

Using the mean score for ranking may allow panel members to “sink” an application by awarding a low score that pulls the mean below the funding line. Including a measure of reviewer disagreement in funding could ameliorate such “sinking” and allow applications that have strong support from a few reviewers to be supported. This may also increase the diversity in what kinds of applications are funded. Some peer review systems already recognise this issue by giving each panel member a wildcard which allows them to “float” an application above the funding line regardless of other panel members’ scores. At least one funding scheme also includes patient and stakeholder reviewers to increase the diversity of viewpoints ^7^.

A recent literature review found “suggestive” evidence that funding peer review can have an anti-innovation bias ^2^, whilst a survey of applicants and reviewers found that innovation and risk may not often be sufficiently addressed in review feedback ^8^. There is evidence that riskier cross-disciplinary research has lower success rates ^9^. Some researchers feel they need to write conservative applications that please all members of the panel to achieve a good mean score ^10^. However, supporting risky research can have huge benefits for society when it pays off ^11^. In a survey of Australian researchers, 90% agreed with the statement: “I think the NHMRC [the main Australian funding body for health and medical research] should fund risky research that might fail but, if successful, would change the scientific field” ^12^.

Previous studies have investigated the association between an application’s mean score (or ranking based on the mean) and subsequent citations, where citations are used as a measure of success. Many studies using large sample sizes found either no association or only a weak association between the mean score and the number of citations of subsequent publications ^13– 17^. Other studies have shown a positive association between better mean peer review scores and increased citations ^18, 19^, including a study that used the same data analyzed here ^20^. To our knowledge, no previous study has empirically estimated how the disagreement in peer reviewers’ scores may also predict citations.

## Methods

### Application data

We examined 227 successful grant applications submitted to the American Institute of Biological Sciences between the years 1999 to 2006. These successful applications came from 2,063 total applications (overall 11% success rate). The applications covered a wide range of biomedical research areas, including vision, drug abuse, nutrition, blood-related cancer, kidney disease, autoimmune diseases, malaria, tuberculosis, osteoporosis, arthritis and autism. Applications were assessed by between 2 and 18 peer reviewers, with a median of 10 reviewers. Panels evaluated an average of 25 applications over two days. Ninety percent of applications were reviewed by on-site panels with an average size of 10 reviewers, and 10% of applications were reviewed via teleconferences of 3 reviewers. Further details on the funding process is available in a previous study of how the applications’ mean scores predicted citations ^20^.

Our key predictor is the peer reviewers’ scores. Individual peer reviewers, who were not conflicted, scored applications between 1.0 (best) to 5.0 (worst) in 0.1 increments. To determine funding, the score was averaged across all reviewers. In this study we also consider statistics that measure within-panel disagreement which are the standard deviation and the range (largest minus smallest score).

### Citation counts

The primary outcome is the citation counts from publications associated with the successful application. The publication data for the funded applications were taken from the mandatory final reports submitted by the applicants. On average, these reports were submitted 5 years after the application’s peer review. Publications were produced from 1 to 8 (average 4.3) years after the review date. Only peer-reviewed publications were counted, confirmed through *PubMed* and *Web of Knowledge* searches. Publications listed in the final report as “submitted” or “in preparation”, were included if they could be found as peer-reviewed published papers. Citations were counted in 2014 using *Web of Knowledge*.

This analysis used 20,313 citations from 805 peer reviewed publications. The total citation level per funded application was the cumulative citations of all publications. As citations are time-dependent they were standardized using the average citation level of all publications by scientific field and year, using data from a published calculation using the *Thomson Reuters Essential Science Indicators database* ^21^. These published average rates were determined for 2000 to 2010 by scientific field, assessed in 2011 and displayed a linear relationship with time (e.g., R ^2^ = 0.99 for the field of molecular biology). We chose molecular biology because it was the highest cited field and in general was the field most applicable to the funded applications.

Because the *Reuters* curve was assessed in 2011, we extended the curve for 2014, back calculating using a linear fit which had a very high R ^2^ of 0.99. In this way, we could most accurately standardize the data for the relationship between publication date and citation level and could calculate the Total Relative Citation per application. A total relative citation of 1 meant the application achieved the average number of citations, whereas values above 1 meant a higher than average number of citations.

We note that a recent study that used both unadjusted citation counts and relative citations, found the two measures gave similar results when used as the key outcome variable ^22^.

### Statistical analyses

To graphically examine associations we used scatterplots of the total relative citations against the application score statistics. If a disagreement in scores indicates a high-risk high-return project, then there may be a greater variation in citations, with more unusually low and high citations for larger disagreements in scores. To examine this we plotted the estimated inter-quartile range in total relative citations by grouping applications using a scatterplot smoothing span ^23^. We used a span of 20 applications which was based on trial and error, and weighted the estimated inter-quartile ranges using a Gaussian kernel ^23^. We used the inter-quartile range instead of the standard deviation because of the strong skew in citations, and the standard deviation was strongly influenced by the application with the highest citations.

### Regression model

The total relative citations were modelled using a multiple regression model. We ran two models with the three application variables:

1. Review year, mean score, score standard deviation
2. Review year, mean score, score range

Our aim was to examine two measures of panel disagreement: the standard deviation and range. We included mean score because it has already been shown to be an important predictor for these data ^20^ and we were interested in the additional value of a measure of disagreement. We adjusted for review year (1999 to 2006) because there was a difference in the application score statistics over time, and because year was associated with citation numbers, hence it was a potential confounder.

The citations were first base e log-transformed because of their positive skew. We added a small positive constant of 0.1 before using the log-transform because some citations were zero. The estimates were back-transformed and plotted to show the results on the original relative citations scale. Using equations the multiple regression model was:

$$\log_{e}(Y_{i}+0.1)\;∼\;N(\mu_{i},\sigma^{2}),\;i=1,...,N,$$

$$\;\mu_{i}\;=\;\beta_{0\;}+\;\sum_{j=1}^{3}\beta_{j}f(X_{i,j})+\gamma_{p(i),}$$

$$\;\gamma_{k}\;∼\;N(0,\sigma_{\gamma }^{2}),\;k=1,...,M,$$

where **Y** are the citations and *γ* are random intercepts to adjust for the potential within-panel correlation in citations (where *p*( *i*) is the panel number for application *i* and *M* is the total number of panels). The mean ( *µ*) had a constant ( *β*_0_) and the three application predictors ( **X**) which were first transformed using a fractional polynomial function.

Associations between the score statistics and citation outcomes could be non-linear, for example a larger difference in citations for a change in mean score from 1.0 to 1.1 compared with a change from 2.0 to 2.1. To model this potential non-linearity we used fractional polynomials to examine a range of non-linear associations between the scores and citations ^24^. The fractional polynomial function is:

$$f(X_{i})\;=\;\{\begin{array}{ll} X_{i}^{p}, & P\neq 0,\\ \log_{e}(X_{i}), & \;P=0. \end{array}$$

We examined the eight transformations of: *P * = {−2, −1, −0.5, 0, 0.5, 1, 2, 3 *}* and chose the optimal *P* using the deviance. The optimal *P* was chosen for each of the three predictors, meaning we examined 8 ^3^ = 512 models in total. We only present results for the best model with the smallest deviance, but the results for the five best deviances are available: https://github.com/agbarnett/funding.disagree.

We checked the distribution of the model residuals for multi-modality and outliers, and used Cook’s distance to find influential observations.

### Missing data

Sixteen (7%) observations were missing the score standard deviation and 32 (14%) observations were missing the score range because the individual peer reviewer scores were no longer available for some applications at the time of this retrospective analysis. These missing observations were imputed using linear regression with the application variables: review year, mean score, score standard deviation, minimum score, maximum score and range. We used five multiple imputations.

All analyses were made using R version 3.4.4 ^25^ with the imputations using the “MICE” package ^26^. The code and anonymized data are available here: http://doi. org/10.5281/zenodo.1452073 ^27^.

We report our results using the STROBE guidelines for observational research ^28^.

## Results

**Figure 1.**
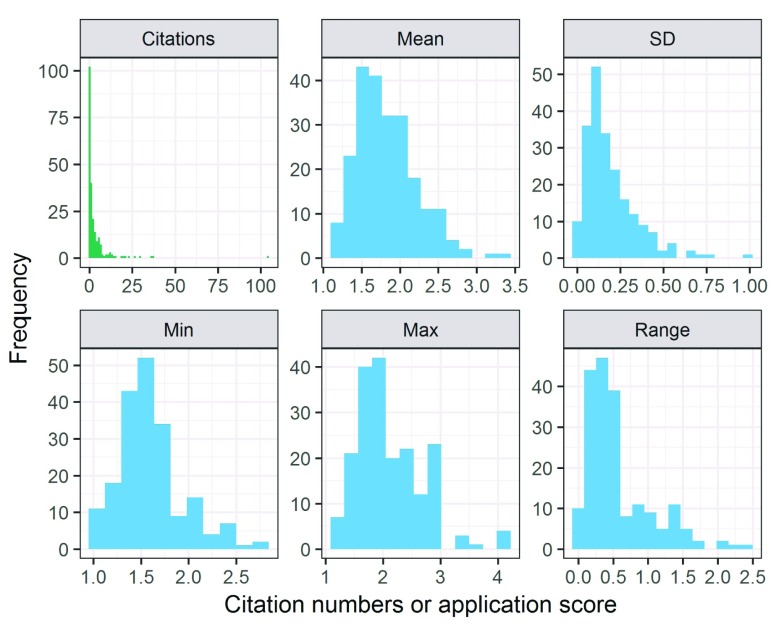
Histograms of total relative citations (green) and application score statistics (blue). The lower the mean score, the better the application did in peer review.

The histograms in Figure 1 show the distributions of total relative citations and the application score statistics: mean, standard deviation, minimum, maximum and range (maximum minus minimum). There was a strong positive skew in citations with one outlying relative citation of 104; the next largest citation was 34. To counter this positive skew we used a base e log transform in later analyses. There was also a positive skew in the score standard deviation and range, and we also log-transform these predictors.

**Figure 2.**
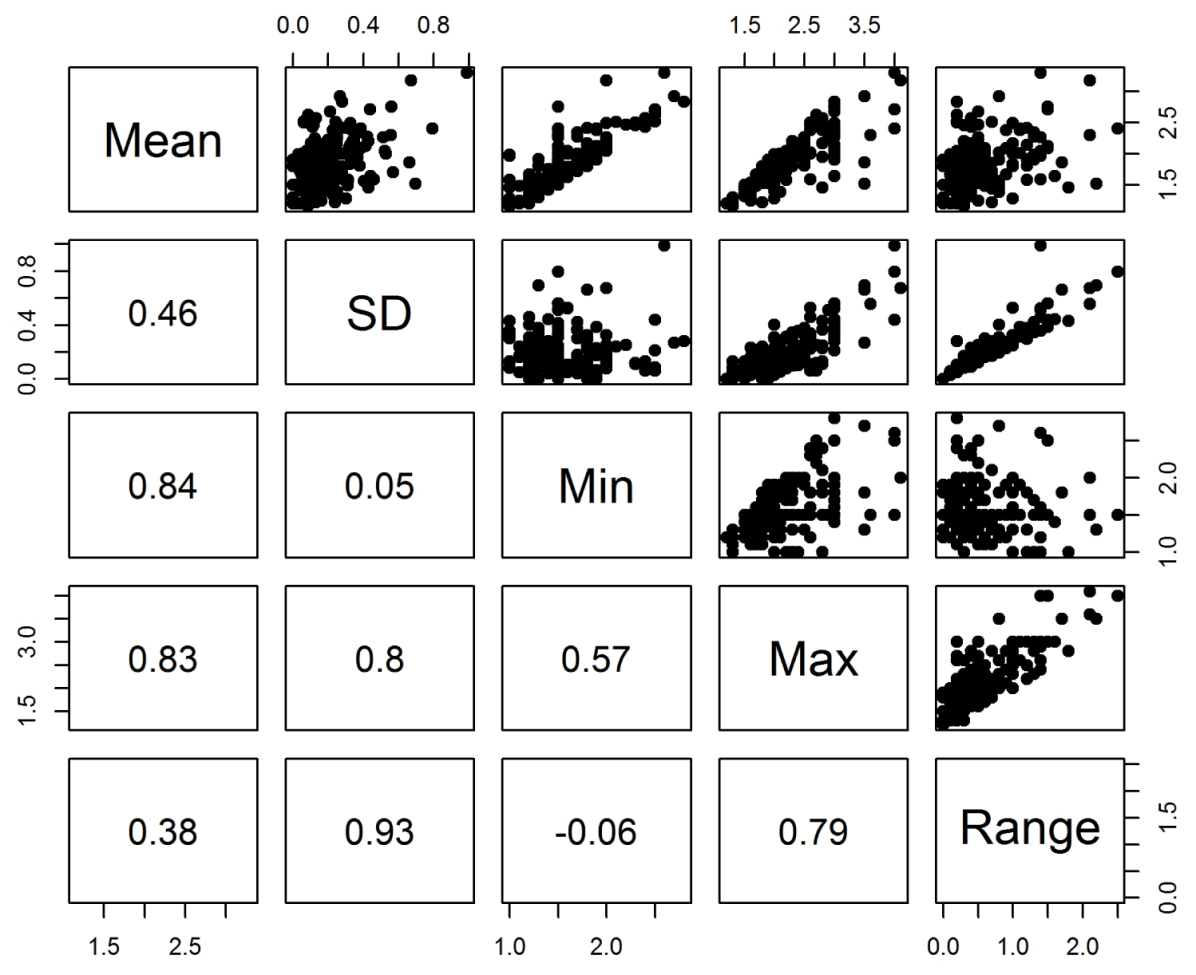
Scatter-plots and Pearson correlations of application score statistics. The numbers in the bottom-left diagonal of the plot matrix are the Pearson correlations.

The scatter-plots in Figure 2 show the associations between the application score statistics. There was a strong correlation between the standard deviation and maximum (0.80), but not between the standard deviation and minimum (0.05). This indicates the largest disagreement is where at least one panel member has given a poor score (remembering that the best possible score is 1.0 and the worst 5.0). Applications where there was one dissenting panel member with a good score were unlikely to be funded as their mean score would not be competitive, and hence are not in this sample. There was a strong positive correlation between the two measures of panel disagreement, the standard deviation and range (0.93).

**Figure 3.**
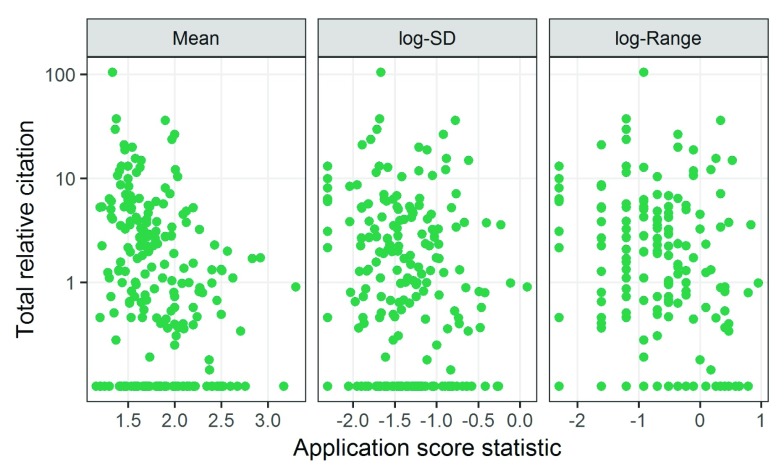
Scatter-plots of log-transformed total relative citations against application score statistics.

The scatter-plots in Figure 3 show the association between total relative citations and the score statistics. We used the log-transformed citations and the standard deviation and range to remove the skew and so show a clearer association. The variance in log-citations appears relatively stable over the score statistics, somewhat confirming the validity of log-transforming the citations ^29^. The points along the bottom of the y-axis are the 74 applications (33%) with no citations. Some association between mean score and citations is visible, with a generally downward pattern in citations for increasing score. There is no clear association between citations and either the standard deviation or range.

**Figure 4.**
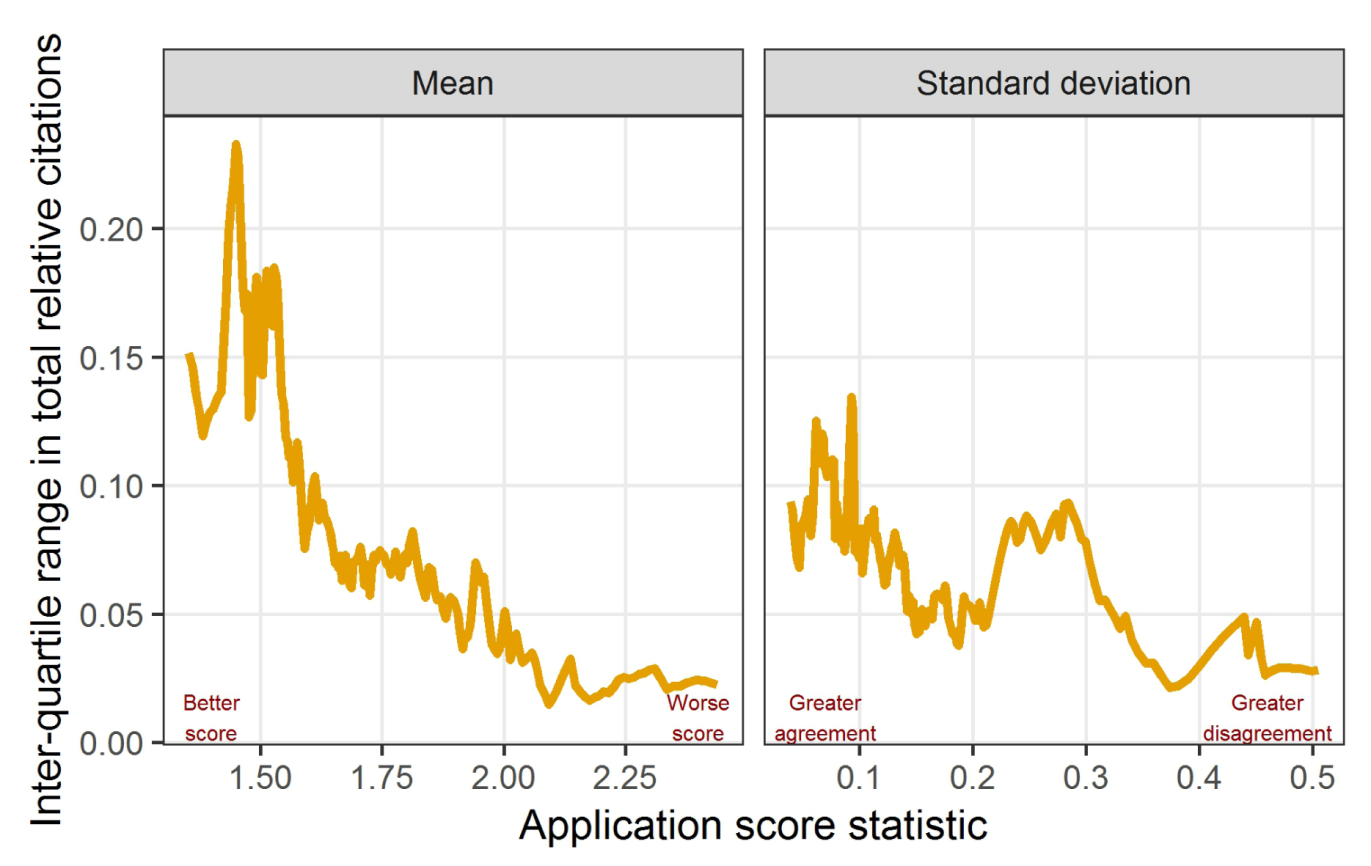
Scatter-plots of the standard deviation in total relative citations against application score statistics.

The inter-quartile ranges in total relative citations by the application scores’ mean and standard deviation are in Figure 4. There was a general reduction in the inter-quartile range as the application score mean increased. The interquartile range also reduced somewhat as the application score standard deviation increased, although the reduction was not as clear as that for the mean.

The results from the multiple linear regression models are in Table 1. Only the application’s mean score had a statistically significant association with citation numbers.

**Table 1.** Parameter estimates for the multiple regression models predicting citation numbers. FP = fractional polynomial.

Model 1	FP	Mean (95% CI)
Mean score	–0.5	5.9 (2.6 to 9.1)
Standard deviation	3	0.7 (–1.3 to 2.7)
Review year	–0.5	–1.2 (–3.0 to 0.6)
Model 2	FP	Mean (95% CI)
Mean score	–0.5	5.4 (2.2 to 8.7)
Range	–0.5	–0.1 (–1.0 to 0.8)
Review year	–0.5	–1.2 (–3.0 to 0.6)

**Figure 5.**
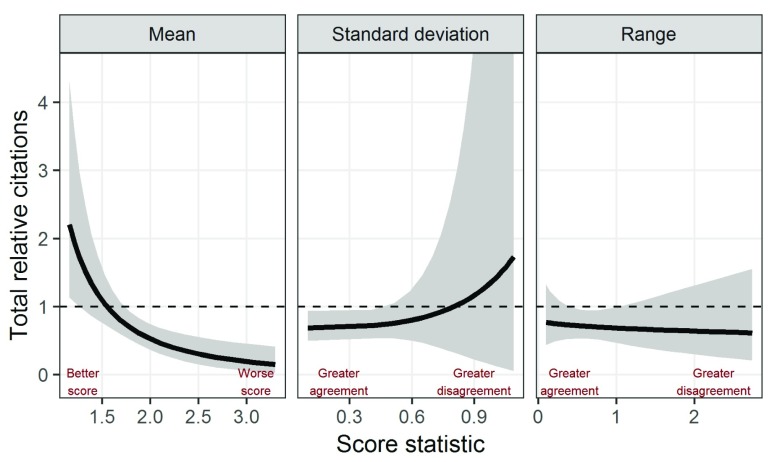
Predicted relative citations using the best fractional polynomial for the application score statistics. The solid lines are the means and the grey areas are 95% confidence intervals. The dotted horizontal line at 1 represents the average citations, so values above this are better than average.

The predictions from the multiple linear regression models are in Figure 5. There was a reduction in citations for applications with a worse mean score. The mean lines are flat for both the standard deviation and range, indicating no association between these score statistics and citation numbers. The 95% confidence intervals for the standard deviation are very wide for large standard deviations. The application with the largest standard deviation was influential according to Cook’s statistic, and removing this application had little impact on the mean line but did reduce these wide intervals (see additional results at https://github.com/agbarnett/funding.disagree). The model residuals had an approximately symmetric and unimodal distribution with no outliers.

## Discussion

We found a statistically significant association between an application’s mean score and subsequent citations, with the result in the expected direction because applications with better scores had more citations (on average). The largest size of the increase is also practically significant as the highest scores have a mean of 2 ( Figure 5), meaning double the average citations.

We found no association between the two measures of reviewer disagreement and citations counts. It appears that any disagreement between peer reviewers did not indicate an application with a potentially high return.

Disagreements between reviewers about an application can stem from different sources. Disagreements about the proposed methods may mean the study is not viable and would struggle to produce valid results and/or publish papers. Disagreements about the application’s goals may reflect a difference of opinion about the potential impact, and it is these disagreements that are likely more subjective and hence where a higher return is possible if one reviewer is right. Disagreements between reviewers can also occur for more trivial reasons such as the dynamics of the panel and personal disagreements ^30^. A more sophisticated measure of panel disagreement to those used here may be more predictive of the benefits of the research, but such measures would need to be well-defined and prospectively recorded at the panel meeting. It may be possible to measure disagreement using an observer who watches the panel dynamics ^30, 31^. Some reviews already breakdown scores into separate areas, such as track record and innovation, and reviewer disagreement could be examined using these separate scores.

Each reviewer brings their own experience and biases to the funding process and such intellectual differences influence application scores ^32, 33^. Indeed, some recent research has indicated that there is more variability in scores across reviewers than across proposals ^34^ and previous studies indicate inter-rater reliability as very low ^35^. An application’s average score is somewhat due to the “luck of the draw” of what reviewers were selected ^36^. Variations can also occur because of the way the application is summarised at the panel meeting ^30, 37^.

If a larger disagreement leads to high-risk returns then we might expect an increase in the variance in citations for larger score standard deviations and ranges. This is because there might be more “failures” with zero citations, but also more big returns. Our multiple regression models only examined a change in mean citations and a different statistical model would be needed to examine a change in variance. However, the scatter-plots in Figure 3 show no sign of an increasing variance in citations for higher standard deviations or ranges, and the inter-quartile ranges in citations in Figure 4 show a slight decrease for greater disagreements.

### Limitations

Some have argued that studies like ours are invalid because: 1) they only consider funded applications and do not include unfunded studies, 2) an application’s score is not the only criteria used to award funding (e.g., applications with low scores awarded funding because of national priorities), and 3) because budgets are frequently cut, meaning the actual research may differ from the application ^38^. Studies that follow funded and unfunded fellowship applicants are possible, e.g., Bornmann et al (2008) ^39^, but this is very difficult when examining projects that need specific funding ^40^. We believe, despite the limitations of bibliometric measures, it is reasonable to expect a dose-response association between scores and citations within funded applications. Samples that include applications that were funded for reasons other than their mean score, such as national priorities, increase the variance in the key predictors of application scores statistics and hence increase statistical power. Cuts to the budget are important and can hinder the planned research. However, assuming the reviewers believed the study was still viable with the reduced budget, such studies still test the ability of a panel to predict what research will have the greatest return.

Citations are an imperfect measure of the impact of research because many citations have little worth and scientists often report that their most highly cited work is not their best ^41^. Studies that examine more detailed outcomes such as translation into practice or cost-benefits would be incredibly useful, however these studies would themselves require funding as it would involve further data collection, analyses and interviews of the applicants.

Our results may not be generalisable to other funding schemes, especially as there are large differences between fields in their perceptions of what makes a good application ^30^. It would be useful to examine whether reviewer disagreement is associated with research impact in other funding schemes. It would also be useful to repeat the study in larger sample sizes, particularly because any conclusions could be influenced by a small proportion of applications that have a very high pay-off.

We only had summary statistics on the application scores and hence we could not examine the distribution of scores to look for interesting patterns such as bimodality in scores, indicating a strong split in the peer review panel.

## Conclusions

We found no association between two measures of reviewer disagreement when assessing an application and the subsequent research impact of that application as measured by citation counts.

## Data availability

The code and anonymized data are available here: https://doi.org/10.5281/zenodo.1452073 ^27^
