# Supplementary material for: Do funding applications where peer reviewers disagree have higher citations? A cross-sectional study
Source: F1000Res. 2018 Oct 15;7:1030. Originally published 2018 Jul 9. [Version 2] doi: 10.12688/f1000research.15479.2 (PMC6171721; doi:10.12688/f1000research.15479.2)
Supplement: Peer review history

# Peer review: Referee response for version 1

**DOI:** 10.5256/f1000research.16873.r37261

**Published:** 2018-09-24

**Authors:** Shahar Avin, Steven Wooding

**Affiliations:** 1 Centre for the Study of Existential Risk, University of Cambridge, Cambridge, UK; 2 Centre for Science and Policy, University of Cambridge, Cambridge, UK

The notion that disagreements amongst reviewers might indicate a promising high-risk/high-reward project certainly has currency amongst those who consider how the system might be improved. In addition to the framework of Linton (2016) ^1^, the idea is also present in the model of Brezis (2007) and is implied by several works on the anti-innovation bias of grant peer review (e.g. Greenberg, 1998 ^2^; Gillies, 2008 ^3^). However, this intuitive notion has yet to be put to the test, until the current study, which is a welcome contribution.

 The experimental design and statistical analysis are compelling for an initial study, though as the authors note further complications could be addressed in later work.

 Given the prevailing policy and academic discussions the negative result is surprising and it rules out some suggested approaches to reform of the grant allocation system; however, the dataset limits the generality of the conclusions that can be drawn (through no fault of the authors) – the key limitation being that only 11% of applications can be analysed (the funded ones), a set that is heavily skewed towards the top scoring applications.

 For example, as shown in Figure 4 there is plenty of potential for SD to be associated with increased citation, but not enough data to tell. The authors note that the application with the largest SD drove this large uncertainty – it could be that this application is an outlier, or it could be that it is typical of applications with large SDs but there are very few of them in the dataset because they tended to fall below the funding line.

 Through its analysis, the study forces a refinement of the question of what sorts of disagreements (in extent and driven by what underlying logics) should be considered useful indicators. We suggest the study raises the following THREE questions for future work:

 Firstly it would be valuable to understand the reasons and logics behind the disagreements that arise between reviewers, from the fact that "disagreements between reviewers about an application can stem from different sources". While the authors suggest addressing this with further information about scores, we can also ask if different score means might indicate different "regimes" for disagreement, e.g. disagreement along the lines of "not sure this will work" or "this goes against received wisdom" at relatively high score means, and disagreements along the lines of "not sure what this is about" or "hasn't this been tried before?" at lower score means. Qualitative research asking reviewers about their scoring behaviour could help understand the reasoning that goes with different scoring.

 Secondly, we could reframe the question to address the lack of information on unfunded applications by asking how can funding best be allocated if the amount of funding available is cut by 50%.

 To test this, we reanalysed the data provided by the authors with the following question: assuming only half the funding was available, which of the following selection methods would perform better?

1. Rank proposals based on their mean scores until funding runs out.
2. Pool proposals based on their mean score into buckets, rounding to the nearest integer. Fund all proposals with a mean of 1 (1.0-1.49, n=45), then from the bucket with a mean of 2 (1.5-2.49, n=114) rank proposals based on their standard deviation and fund until funding runs out.

 Under both methods, all proposals with a mean of 1 are funded, for a total TRC of 319.9. When we look at the proposals in the second bucket, when funding according to method 1, the total TRC for this bucket is 224.2, whereas for method 2 it is 195.5. However, the highest citation-receiving proposal in the second bucket under method 1 has a TRC of 19.9, whereas the highest citation-receiving proposal from the second bucket under method 2 has a TRC of 35.9 (the third highest TRC in the entire sample). This fits with Gillies' description of peer-review as going for less risky proposals, but at the cost of throwing out the occasional exceptional proposal. This is also born out by the distribution of TRCs, with the standard deviation of TRCs for the second bucket under method 1 being 4.2, and under method 2 being 6.2.

 Thirdly, it would be valuable to understand what sorts of disagreement exist in the scoring of all the applications (both successful and unsuccessful). It could be argued that the type of disagreement likely to indicate high risk/high return proposals would be bi-modal – some good reviews maybe with scores of 1-1.5, some very poor reviews maybe with scores of 3-4. Given the small fraction of applications funded, very few applications of this nature would be funded (due to the understandable aggregation in the shared data we could not examine the exact number, but fewer than 10 applications have a score lower than 3). Do such applications with such bi-modal review scores exist? Or is the level of disagreement seen in the successful applications similar to that of the unsuccessful applications?

 The above questions only suggest that there is further complexity here that needs to be explored, with more complex, higher power studies. We thank the authors for paving the path for such studies, and for making their underlying data and analysis available in a form that is easy to explore.

 Two small notes:

1. It might be easier to read the paper if scores were consistently referred to as best/worst and better/worse – using ‘higher' is confusing when better scores are lower.
2. It would be more elegant to give 'e.g., Bornmann et al, 2008' rather than 'e.g., 36'.

We have read this submission. We believe that we have an appropriate level of expertise to confirm that it is of an acceptable scientific standard.

## References

[1] LintonJ: Improving the Peer review process: Capturing more information and enabling high-risk/high-return research. *Research Policy*.2016;45(9) : 10.1016/j.respol.2016.07.004 1936-1938 10.1016/j.respol.2016.07.004

[2] GreenbergD: Chance and grants. *The Lancet*.1998;351(9103) : 10.1016/S0140-6736(05)78485-3 10.1016/S0140-6736(05)78485-3 9500372

[3] GilliesD: How should research be organised?. *College Publications*.2008;

# Response

**Published:** 2018-10-10

**Authors:** Adrian Barnett

**Affiliations:** Queensland University of Technology, Australia

*The notion that disagreements amongst reviewers might indicate a promising high-risk/high-reward project certainly has currency amongst those who consider how the system might be improved. In addition to the framework of Linton (2016)1, the idea is also present in the model of Brezis (2007) and is implied by several works on the anti-innovation bias of grant peer review (e.g. Greenberg, 1998 2; Gillies, 2008 3). However, this intuitive notion has yet to be put to the test, until the current study, which is a welcome contribution.*

 Thanks for the interesting references, we have now mentioned Brezis in our Introduction.

*The experimental design and statistical analysis are compelling for an initial study, though as the authors note further complications could be addressed in later work.*

*Given the prevailing policy and academic discussions the negative result is surprising and it rules out some suggested approaches to reform of the grant allocation system; however, the dataset limits the generality of the conclusions that can be drawn (through no fault of the authors) – the key limitation being that only 11% of applications can be analysed (the funded ones), a set that is heavily skewed towards the top scoring applications.*

 We agree this is a key limitation, and it would be useful to repeat the study in a more generous scheme, where a greater proportion of applications were funded.

*For example, as shown in Figure 4 there is plenty of potential for SD to be associated with increased citation, but not enough data to tell. The authors note that the application with the largest SD drove this large uncertainty – it could be that this application is an outlier, or it could be that it is typical of applications with large SDs but there are very few of them in the dataset because they tended to fall below the funding line.*

 This is entirely possible, and if there are a small number of extremely high pay-off projects then we would need a much larger sample to robustly test if there is a consistent pattern of more high pay-off projects associated with greater disagreement. We have now mentioned this in the limitations.

*Through its analysis, the study forces a refinement of the question of what sorts of disagreements (in extent and driven by what underlying logics) should be considered useful indicators. We suggest the study raises the following THREE questions for future work:*

*Firstly it would be valuable to understand the reasons and logics behind the disagreements that arise between reviewers, from the fact that "disagreements between reviewers about an application can stem from different sources". While the authors suggest addressing this with further information about scores, we can also ask if different score means might indicate different "regimes" for disagreement, e.g. disagreement along the lines of "not sure this will work" or "this goes against received wisdom" at relatively high score means, and disagreements along the lines of "not sure what this is about" or "hasn't this been tried before?" at lower score means. Qualitative research asking reviewers about their scoring behaviour could help understand the reasoning that goes with different scoring.*

 This is a good point. “Disagreement” is likely to be too general a word, and future studies could look in far more detail at the types of disagreement. We have previously used a qualitative researcher as an observer of funding panel dynamics, and we have now added this potential approach to our discussion.

*Secondly, we could reframe the question to address the lack of information on unfunded applications by asking how can funding best be allocated if the amount of funding available is cut by 50%.*

*To test this, we reanalysed the data provided by the authors with the following question: assuming only half the funding was available, which of the following selection methods would perform better?*

*Rank proposals based on their mean scores until funding runs out.*

*Pool proposals based on their mean score into buckets, rounding to the nearest integer. Fund all proposals with a mean of 1 (1.0-1.49, n=45), then from the bucket with a mean of 2 (1.5-2.49, n=114) rank proposals based on their standard deviation and fund until funding runs out.*

*Under both methods, all proposals with a mean of 1 are funded, for a total TRC of 319.9. When we look at the proposals in the second bucket, when funding according to method 1, the total TRC for this bucket is 224.2, whereas for method 2 it is 195.5. However, the highest citation-receiving proposal in the second bucket under method 1 has a TRC of 19.9, whereas the highest citation-receiving proposal from the second bucket under method 2 has a TRC of 35.9 (the third highest TRC in the entire sample). This fits with Gillies' description of peer-review as going for less risky proposals, but at the cost of throwing out the occasional exceptional proposal. This is also born out by the distribution of TRCs, with the standard deviation of TRCs for the second bucket under method 1 being 4.2, and under method 2 being 6.2.*

 This is an interesting comment and thanks for the new analysis. As an aside, we note that only by sharing our data can we have such an in-depth discussion with our reviewers. We have added R code to run these suggested analyses to our results on github ( https://github.com/agbarnett/funding.disagree).

 It is an interesting approach to use the mean for the “top” tier of applications and the standard deviation for the second tier. This may appeal to funders and panel members, because we believe there will always be a desire to fund those applications with the best mean. This two-tier approach may also be easier to explain than the Black–Scholes approach suggested by Linton, which combines the mean and variance in scores using an equation.

 We are wary of using the standard deviation in the total relative citations because of the strong positive skew in its distribution, and the inter-quartile range in citations is actually larger for the approach using the mean at 4.3, compared with 2.9 when using the standard deviation.

*Thirdly, it would be valuable to understand what sorts of disagreement exist in the scoring of all the applications (both successful and unsuccessful). It could be argued that the type of disagreement likely to indicate high risk/high return proposals would be bi-modal – some good reviews maybe with scores of 1-1.5, some very poor reviews maybe with scores of 3-4. Given the small fraction of applications funded, very few applications of this nature would be funded (due to the understandable aggregation in the shared data we could not examine the exact number, but fewer than 10 applications have a score lower than 3). Do such applications with such bi-modal review scores exist? Or is the level of disagreement seen in the successful applications similar to that of the unsuccessful applications?*

 Unfortunately our data only contains the summary statistics on the applications’ scores and so we can’t examine these distributions. We have added this as a limitation.

*The above questions only suggest that there is further complexity here that needs to be explored, with more complex, higher power studies. We thank the authors for paving the path for such studies, and for making their underlying data and analysis available in a form that is easy to explore.*

 Thanks, we agree that larger studies are needed.

*Two small notes:*

- *It might be easier to read the paper if scores were consistently referred to as best/worst and better/worse – using ‘higher' is confusing when better scores are lower.*

Agreed and changed. We’ve also added text labels to Figure 5 and the new Figure 4.

- *It would be more elegant to give 'e.g., Bornmann et al, 2008' rather than 'e.g., 36'.*

Changed as suggested.

# Peer review: Referee response for version 1

**DOI:** 10.5256/f1000research.16873.r36742

**Published:** 2018-09-20

**Authors:** Jonathan Shepherd, Jeremy C Wyatt

**Affiliations:** 1 Southampton Health Technology Assessments Centre, University of Southampton, Southampton, UK; 2 Wessex Institute, University of Southampton, Southampton, SO40 7AA, UK

We thank the authors for this very interesting study. We have some comments and suggestions which we think will enhance the manuscript.

 1. The primary outcome measure is the citation counts from publications associated with the successful application. Publications were produced from 1 to 8 years after the peer review date (average 4.3 years). This does not appear to take account of varying time since the projects were funded (i.e. 7 year gap between projects that were funded from 1999 to 2006). Thus, older studies would have had more time for publications to be produced and cited. We therefore suggest a more meaningful outcome measure would be either the number of citations per year per study, or the total number of citations in, say, the 5 years following the final project report, or some other standardised project milestone. Adding the review year to the model does not seem to adequately control for this factor (though qualified statistical advice is needed to clarify this). We think this is the issue that likely affects the interpretation of the results the most in our critique. 

 2. The number of citations was standardised by academic field – i.e. molecular biology. Was there any variation in study designs within this field that might also change the expected number of citations (e.g. systematic reviews may attract more citations than primary experimental studies in some fields)? It would be useful for context if there could be a table with some basic aggregate details of the funded studies, such as types of study design, molecular biological application, study sample characteristics, duration of study etc. This would help to put the results into context. 

 3. The impact of a piece of research on which referees could not agree might be either lower or higher than those on which they could. So, it would be useful to plot the standard deviation of the citations against the standard deviation of the peer review score. 

 4. As well as using multiple imputation to correct for missing data, a sensitivity analysis in which cases with missing data are omitted would be useful. 

 5. Some measure of the model fit would be useful, eg. adjusted R squared 

 6. There was a wide variation in the number of reviewers per article from 2 to 18. Was this due to differences in the kind of research, amount of funding requested or some other perceived risk on behalf of the funder? Could this artificially influence the standard deviation of the score, confounding any association with citations? 

 7. Fractional polynomial model results are only presented for the best fitting model with the smallest deviance. This is acceptable in principle, but it would be useful for the authors to comment on whether there was any variation in the results according to the other fractional polynomial transformations (if available). This will provide confidence in the robustness of the findings. 

 8. Reference is made in the first paragraph to a “recent systematic review” by Guthrie et al (2018 ^1^) (and also in the third paragraph). We note that this publication doesn’t refer to itself as being a systematic review, and indeed, it is an update of a 2009 review which describes itself as a non-systematic review. We would suggest using the tern “non-systematic review”, or just “literature review”. 

 9. Thank you for citing our own recent systematic review on peer review of grants in health. You mention that the review included eight studies and called for further research in this area, which is correct. However, the review focused specifically on studies aiming to improve the effectiveness and efficiency of peer review. These were drawn from a wider set of 83 studies on peer review which we systematically mapped. In the map there were some studies which focused on assessing the impact of funded research, eg. In terms of bibliometrics. Thus, there is a body of evidence on this topic, though we didn't systematically review it in detail. We are happy to provide you with a list of these studies. 

 10. The sentence on page 3 beginning “A recent systematic review found “suggestive” evidence that funding peer review can have an anti-innovation bias ^2^ and that innovation and risk may not often be sufficiently addressed in review feedback ^7^” needs re-wording as not only is the Guthrie et al paper a non-systematic review, but the second reference cited in that sentence by Gallo et al is a survey (i.e. not a review at all). The way the sentence is phrased implies that it is a systematic review. 

 11. Suggest amending the sentence on page 3 “Many studies using large sample sizes found either no association or only a weak association between the mean score and the VOLUME of citations of subsequent publications”

We have read this submission. We believe that we have an appropriate level of expertise to confirm that it is of an acceptable scientific standard.

## References

[1] GuthrieSGhigaIWoodingS: What do we know about grant peer review in the health sciences?. *F1000Res*.2017;6: 10.12688/f1000research.11917.2 1335 10.12688/f1000research.11917.2 29707193PMC5883382

# Response

**Published:** 2018-10-10

**Authors:** Adrian Barnett

**Affiliations:** Queensland University of Technology, Australia

*1.    The primary outcome measure is the citation counts from publications associated with the successful application. Publications were produced from 1 to 8 years after the peer review date (average 4.3 years). This does not appear to take account of varying time since the projects were funded (i.e. 7 year gap between projects that were funded from 1999 to 2006). Thus, older studies would have had more time for publications to be produced and cited. We therefore suggest a more meaningful outcome measure would be either the number of citations per year per study, or the total number of citations in, say, the 5 years following the final project report, or some other standardized project milestone. Adding the review year to the model does not seem to adequately control for this factor (though qualified statistical advice is needed to clarify this). We think this is the issue that likely affects the interpretation of the results the most in our critique.*

Thanks for the comment. Actually, the citation values are normalized per publication year, thus older studies would not have an advantage as the resultant citations from publications are normalized by year. It is true that more time post peer review date would allow more publications, but all of these products were derived from the final reports, which were submitted on average five years after peer review. Thus, whatever is reported by the principal investigator in their final report is what was measured, which is what is presented by funding agencies as the output of the grant.

*2. The number of citations was standardised by academic field – i.e. molecular biology. Was there any variation in study designs within this field that might also change the expected number of citations (e.g. systematic reviews may attract more citations than primary experimental studies in some fields)? It would be useful for context if there could be a table with some basic aggregate details of the funded studies, such as types of study design, molecular biological application, study sample characteristics, duration of study etc. This would help to put the results into context.*

This is interesting, but would likely need to be a much larger, separate study to go back into each application and characterize the study design, sample characteristics, etc., and is out of the current scope. That said, the applications included in this analysis were submitted to a 4-year, R01-style support mechanism. In every program year there was a significant proportion of both applied and basic research applications, with many applications encompassing varying degrees of both basic and applied research in their aims. There was a wide variety of research topic areas, including vision, drug abuse, nutrition, blood-related cancer, kidney disease, autoimmune diseases, malaria, tuberculosis, osteoporosis, arthritis, and autism research, among others. This is described in more detail in our original publication: https://journals.plos.org/plosone/article?id=10.1371/journal.pone.0106474.

*3. The impact of a piece of research on which referees could not agree might be either lower or higher than those on which they could. So, it would be useful to plot the standard deviation of the citations against the standard deviation of the peer review score.*

We have now plotted the inter-quartile range in citations against the application score mean and standard deviation. We used the inter-quartile range instead of the standard deviation because of the strong skew in citations, and the standard deviation was strongly influenced by the application with the highest citations.

The new results show moderate support for a greater return for applications with a higher agreement between peer reviewers. See new Figure 4, with new explanatory paragraph in the “Statistical Methods” section, and an updated discussion.

*4. As well as using multiple imputation to correct for missing data, a sensitivity analysis in which cases with missing data are omitted would be useful.*

We have added a complete case analysis to the more detailed results available on github ( https://github.com/agbarnett/funding.disagree). All the results were very similar to the imputed data.

*5. Some measure of the model fit would be useful, eg. adjusted R squared*

We are not certain that the R-squared statistic would be useful here. The adjusted R-squared can be useful for comparing between alternative models, or where the goal is accurate prediction. Here we were just looking for any signal between application scores and citations, assuming that the detection of any signal could lead to improvements in the design of funding systems. We have investigated the residuals to look for poor model fit.

*6. There was a wide variation in the number of reviewers per article from 2 to 18. Was this due to differences in the kind of research, amount of funding requested or some other perceived risk on behalf of the funder? Could this artificially influence the standard deviation of the score, confounding any association with citations?*

The majority (90%) of applications were reviewed in panels (roughly 10 to 15 reviewers). Some were reviewed via a mail review mechanism (2 to 3 reviewers). The determination of review mechanism largely depended on the topic area. We don’t believe this should be a large confounder of the results.

We have now included a sensitivity analysis using just the larger panels and excluding the mail review panels. The results were similar to the analysis using all panels. These new results are available on github ( https://github.com/agbarnett/funding.disagree).

*7. Fractional polynomial model results are only presented for the best fitting model with the smallest deviance. This is acceptable in principle, but it would be useful for the authors to comment on whether there was any variation in the results according to the other fractional polynomial transformations (if available). This will provide confidence in the robustness of the findings.*

We have examined the predictions for the best five models and the average results were similar in terms of the mean. However, the confidence intervals for the second best model had a much narrow confidence interval for citation predictions using the application score standard deviation. In hindsight we prefer this model, as it has only a slightly poorer deviance, but a much more believable confidence interval. It would have been better to have a multi-criteria decision for the “best” model, that used both the deviance and average confidence interval width. This has been a useful lesson for future studies, but we continue to present the model with the wider CI in the main results because we feel we should stick to our original protocol.

These new results are available on github ( https://github.com/agbarnett/funding.disagree).

*8. Reference is made in the first paragraph to a “recent systematic review” by Guthrie et al (2018 1) (and also in the third paragraph). We note that this publication doesn’t refer to itself as being a systematic review, and indeed, it is an update of a 2009 review which describes itself as a non-systematic review. We would suggest using the term “non-systematic review”, or just “literature review”.*

Thank you for flagging this. We have now used “literature review”.

*9. Thank you for citing our own recent systematic review on peer review of grants in health. You mention that the review included eight studies and called for further research in this area, which is correct. However, the review focused specifically on studies aiming to improve the effectiveness and efficiency of peer review. These were drawn from a wider set of 83 studies on peer review which we systematically mapped. In the map there were some studies which focused on assessing the impact of funded research, eg. In terms of bibliometrics. Thus, there is a body of evidence on this topic, though we didn't systematically review it in detail. We are happy to provide you with a list of these studies.*

We have changed our language to make the goal of your review clearer. We would gladly see the study list. Perhaps this is best uploaded to our github page for this project (if it can be made public): https://github.com/agbarnett/funding.disagree then click “upload file”, you will need a github account (free) and request write access to our project.

*10. The sentence on page 3 beginning “A recent systematic review found “suggestive” evidence that funding peer review can have an anti-innovation bias2 and that innovation and risk may not often be sufficiently addressed in review feedback7” needs re-wording as not only is the Guthrie et al paper a non-systematic review, but the second reference cited in that sentence by Gallo et al is a survey (i.e. not a review at all). The way the sentence is phrased implies that it is a systematic review.*

Thank you for flagging this. We have now used “literature review” and added the fact that the second study is a survey.

*11. Suggest amending the sentence on page 3 “Many studies using large sample sizes found either no association or only a weak association between the mean score and the VOLUME of citations of subsequent publications”*

We have changed the text to read “number of citations”.
